# Impact of waste management training intervention on knowledge, attitude and practices of teaching hospital workers in Pakistan

**DOI:** 10.12669/pjms.323.9903

**Published:** 2016

**Authors:** Ramesh Kumar, Ratana Somrongthong, Jamil Ahmed

**Affiliations:** 1 Ramesh Kumar, MBBS, PhD. College of Public Health Sciences, Chulalongkorn University, Bangkok, Thailand, Assistant Professor Health Services Academy Islamabad, Pakistan; 2 Ratana Somrongthong, PhD. Associate Professor College of Public Health Sciences, Chulalongkorn University, Bangkok, Thailand; 3 Jamil Ahmed, FCPS. Department of Family and Community Medicine, College of Medicine and Medical Sciences, Arabian Gulf University, Bahrain

**Keywords:** Hospital workers, Intervention, Knowledge attitude and practice, Training, Waste management

## Abstract

**Objective:**

To evaluate the sustainability and effectiveness of training as an intervention to improve the knowledge, attitude and practices of hospital workers on health care waste management.

**Method:**

We conducted this quasi-experimental study in two tertiary care teaching hospitals in Rawalpindi in October 2013. Training, practical demonstrations and reminders on standard waste management were given to 138 hospital workers in one hospital and compared with 137 workers from the control hospital. We collected data 18 months after intervention through a structured questionnaire to assess the impact of the intervention. We used paired t-test to compare the scores on knowledge, attitude and practices at baseline and first follow up and final impact assessment. Chi square test was used to compare group variables between intervention and control groups.

**Results:**

After 18 months since intervention the mean scores on knowledge attitude and practices differed statistically significantly since baseline and intervention group had statistically significantly better knowledge positive attitudes and good health care waste management practices (p < 0.001). Health care and sanitary workers in intervention group scored statistically significantly higher (p < 0.001).

**Conclusion:**

Trainings of health and sanitary workers on health care waste management guidelines were sustainable among the intervention group after 18 months which shows the positive impact of our intervention. It is recommended that the trainings as intervention be included in the overall policies of the public and private sector hospitals in Pakistan and other similar settings.

## INTRODUCTION

Waste management is a challenge in hospitals of Lower middle income countries (LMIC) resulting in high burden of hospital acquired infections.^1^ These countries suffer from improper infectious waste management practices in their hospitals that causes an occupational and public health challenge for the general public.^2^ Health care activities in these hospitals produce infectious waste which may cause a higher risk for infection and injury than any other type of waste. Mishandling of health care waste due to lack of knowledge of healthcare workers (HCWs) may have serious effects on the environment and health. Hospitals generate about 0.5 to 2.0 kg of waste per bed per day; globally.^3^ About 75-90% of this waste is non-infectious while 10-25% is infectious. Non-infectious waste consists of non-hazardous materials which do not have any potentially harmful effects on health and do not need any special management and disposal measures. Infectious waste is composed of potentially hazardous materials such as sharps, syringes, needles, blades, instruments, human tissues and parts, waste contaminated with blood, body secretion and vomitus of the patients and other contagious and infectious items. This waste needs to be disposed of properly by the trained personnel.^4^

Reuse of the syringes is another serious public health problem reported globally, resulting in potential threats to the general public. The main threat is needle prick injuries especially among HCWs.^5^ Particularly, these sharp instruments have reported frequent injuries among the health care workers.^6^ Infectious waste results in severe health problems due to their highly infectious nature because that contains toxic substances such as microorganisms and chemicals.^7^ Approximately 12,000 million injections are used every year and constitute about 1% of sharp waste globally. In Pakistan 52% of doctors suffer needle prick injuries in hospitals.^8^ Infectious Health care waste is composed of the materials that are produced from medical treatment in the medical units such as offices of general practitioners, dental clinics, chiropractors, acupuncture, at home patient care, from harm reduction programs for drug addicts, maternity homes, diagnostics laboratories, immunization centers and scientific research institutions.^9^ Mismanagement of infectious health care waste results in environmental pollution and unpleasant odors due to harmful pathogens that may develop many infections such as typhoid, cholera, tuberculosis and other diseases namely hepatitis and HIV/AIDS. Health workers, patients, waste handlers, waste pickers and general masses are prone to acquire these infections. Hence, there is an urgent need to have all kinds of wastes be treated properly.^10^

Hospitals in Pakistan produce about 1.35 kg of health care waste every day per bed. There are around 92,000 beds available in the public sector hospitals of Pakistan which produce 0.8 million tons of waste each day.^11^ A large amount of health care waste is incinerated but this practice is limited due to the environmental concern because burning of solid and health care waste produced by health facilities may result in environmental health problems. Health care waste incinerators discharge toxic smoke and poisonous ash residues which are source of dioxins in the environment. The noxious ash residues are finally disposed off in the landfill sites which ultimately are converted as a leach into groundwater and contaminate it. Health care wastes has been recognized by the environmental agency in the USA as the 3rd leading cause of dioxin air pollution and add to 10% of mercury poisoning in the environment from human activities.^12^ Dioxin is known to be lethal toxic chemicals which affects human health very badly and causes cancer, immune system disorders, Diabetes Mellitus, birth defects and interrupts the reproductivedevelopment.^13^

Trainings of health workers have been proven to be one of the most effective strategies for improving the practices and health behavior, especially when combined with other innovativeapproaches.^14^-^15^ It has been shown that regular trainings of healthcare workers could improve their practices of waste management at their work places.^16^ Trainings of healthcare workers are essential to improve their behavior towards hospital waste management.^17^ We implemented the training model for six months of time and checked their effectiveness after the successful intervention and it proved to be effective.^16^ However the studies on the sustainability and impact of this kind of training model are scarcely available. Hence, objectives of this study was to measure the long term impact and sustainability of such an intervention on the knowledge, attitude and practices of hospital workers to handle HCW based on standard procedures.

## METHODS

We used a quasi-experimental design in this study and intervention was carried out in one tertiary care teaching hospital (Holy Family) in the city of Rawalpindi in October 2013. Another similar size hospital was taken as a control. Detailed methodology for the research is explained elsewhere.^16^ In brief, for the current study we collected data after 18 months of intervention through a structured questionnaire to assess the impact of the intervention since the baseline data and first follow up done at three months after the intervention.

**Fig.1:**
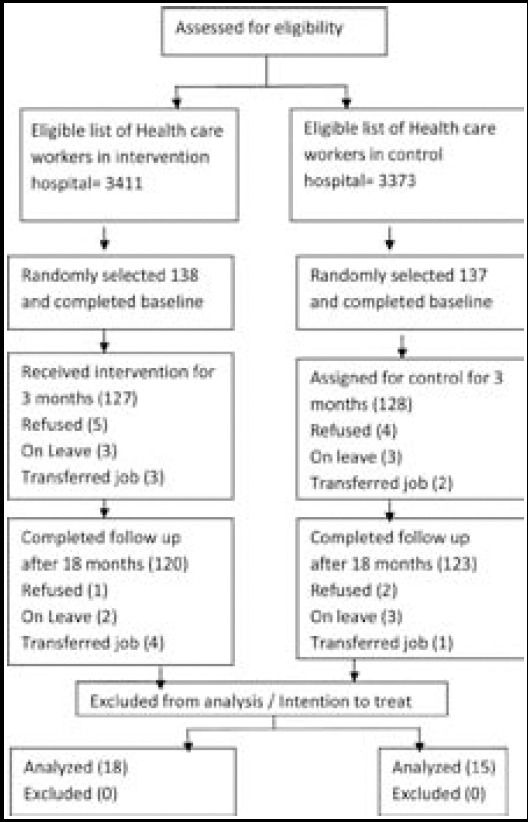
Study allocation and evaluation.

We selected 138 and 137 workers (health and sanitary) from intervention and control hospitals, respectively, through simple random sampling (Fig.1). We used paired t test to compare the knowledge, attitude and practice composite scores at baseline, first follow-up and at the final assessment to measure the impact. We also used chi square test to measure the statistical differences between the intervention and control groups in the study with regard to grouped variables on Knowledge, attitude and practice. Study was approved by the Intuitional review board of Health Services Academy; Pakistan (F.No.3-107/2013-IERC/HSA); while consent was also taken from both participants.

## RESULTS

### Health workers

We ran paired t-test on a sample of 101 health workers in the intervention group to determine whether there was a statistically significant mean difference between the knowledge, attitude and practice scores about HCWM at Baseline and first follow-up and compared to scores at final follow-up to measure the impact of the intervention over time. We found that participants had statistically significantly (P<0.05) better knowledge, attitude and practices at final follow up as opposed to baseline with a mean difference of 5.7±2.7, 6.5±9.3 and 3.5±4.3 respectively. There was no statistically significant change in scores from first follow up to final follow up with respect to Knowledge, attitude or practice (Table-I).

**Table-I:** Paired t-test Attitude, knowledge and practice scores at final follow-up compared with baseline and first follow-up in health workers (n=101).

		*Mean score±SD*	*Mean Difference inscore±SD*	*95% C.I. of difference*		*P value*

				*Lower*	*Upper*	
Pair 1	Knowledge at final follow-up	18.6±2.2	5.7±2.7	5.17	6.24	<0.001
	Knowledge at Baseline	12.9±3.1				
Pair 2	Knowledge at final follow-up	18.6±2.2^a^				
	Knowledge at first follow-up	18.6±2.2^a^				
Pair 3	Attitude final follow-up	34.1±4.1	6.5±9.3	4.7	8.3	<0.001
	Attitude at Baseline	27.6±7.3				
Pair 4	Attitude at final follow-up	34.1±4^a^				
	Attitude at first follow-up	34.1±4^a^				
Pair 5	Practice at final follow-up	14.8±2.5	3.5±4.3	2.64	4.36	<0.001
	Practice at Baseline	11.3±3.9				
Pair 6	Practice at final follow-up	14.8±2.5^a^				
	Practice at first follow-up	14.8±2.5^a^				

a The correlation and t cannot be computed because the standard error of the difference is 0.

Comparison of composite variables of Knowledge attitude and practices in health workers by using chi square is shown in Table-II. We found that knowledge was statistically significantly higher, attitudes positive and practices were good in health workers at the final follow up or during the impact assessment (P=<0.001).

**Table-II:** Knowledge, Attitude and practices of control and intervention groups in health staff at the final follow-up in health workers (n=203).

	*Control (n=102) n (%)*	*Intervention (n=101) n (%)*	*P-Value*
*Knowledge at final follow-up*			
Low	8 (8)	0 (0)	<0.001
Intermediate	88 (86)	30 (30)	
High	6 (6)	71 (70)	
*Attitude at final follow-up*			
Positive	44 (43)	83(82)	<0.001
Negative	58 (57)	18 (18)	
*Practice at final follow-up*			
Good	63 (62)	88 (87)	<0.001
Bad	39 (38)	13 (13)	

### Sanitary workers

In the paired t-test analysis of a sample of 52 sanitary workers to determine whether there was a statistically significant mean difference between the knowledge, attitude and practice scores about HCWM at baseline and first follow-up and compared to scores at final follow-up to measure the impact of the intervention over time. We found that sanitary workers had statistically significantly (P<0.05) better knowledge about HCWM at final follow up as opposed to baseline with a mean difference 4.6±2.3 points; they also fared better in attitudes and practices about HCWM at final follow up as opposed to the baseline scores with mean difference of 4.0 ±8.3 and 6.7±5.1 points, respectively. There was no statistically significant change in sanitary workers’ knowledge, attitude and practices about HCWM from first to final follow up (Table-III).

**Table-III:** Paired t-test of Attitude, knowledge and practice scores at final follow-up compared with baseline and first follow-up in intervention group of sanitary workers (n=26).

		*Mean±SD*	*Mean Diff±SD*	*95% C.I. of difference*		*P-value*

				*Lower*	*Upper*	
Pair 1	Knowledge at final follow-up	12.9±3.0	4.6±2.3	3.68	5.62	<0.001
	Knowledge at Baseline	8.3±3.1				
Pair 2	Knowledge at final follow-up	12.9±3.0^a^				
	Knowledge at first follow-up	12.9±3.0^a^				
Pair 3	Attitude final follow-up	31.8±4.9	4.0 ±8.3	0.67	7.40	0.021
	Attitude at Baseline	27.8± 8.7				
Pair 4	Attitude at final follow-up	31.8±4.9^a^				
	Attitude at first follow-up	31.8±4.9^a^				
Pair 5	Practice at final follow-up	9.2±3.0	6.7±5.1	4.65	8.80	<0.001
	Practice at Baseline	2.5±3.8				
Pair 6	Practice at final follow-up	9.2±3.0^a^				
	Practice at first follow-up	9.2±3.0^a^				

a The correlation and t cannot be computed because the standard error of the difference is 0.

Cross-tabulation obtained through chi-square test and comparison of composite variables in sanitary workers showed that knowledge was statistically significantly higher, attitudes positive and practices were good in health workers at the final follow up or during the impact assessment (P=<0.001, Table-IV).

**Table-IV:** Chi Square test of significance of Knowledge, attitude and practices in sanitary workers at the final follow-up (n=52).

	*Control(n=26) n (%)*	*Intervention(n=26) n (%)*	*P*
*Knowledge at final follow-up*			
Low	11(42%)	1(4%)	<0.001
Intermediate	15(58%)	12(46%)	
High	0(0%)	13(50%)	
*Attitude at final follow-up*			
Positive	23(88%)	11(42%)	0.01
Negative	3(12%)	15(58%)	
*Practices at final follow-up*			
Good	24(92%)	16(62%)	0.009
Bad	2(8%)	10(38%)	

## DISCUSSION

In this study we assessed the impact of our intervention which required the passage of adequate time. We focused to determine if the effects training of health and sanitary workers on HCWM guidelines and procedures were sustainable after a sufficient time period had elapsed. We found that levels of knowledge, attitude and practices remained higher than their controls. These statistically significant differences persisted after a period of one and a half year after training. Therefore our results contribute toward and build upon the current evidence on the sustainability and usefulness of trainings as a one of the key interventions for the improvement of and adherence to the waste management guidelines in health care sector. In addition to this, our study results stand on a unique methodology which used extended trainings, practical demonstrations and reminders on infectious waste management for the intervention group.

Our results in the current study confirm and extend the evidence generated which showed the effectiveness of the trainings of healthcare workers measured after six months.^16^ Hospital workers in developing countries generally lack required knowledge and skill to practice optimum waste management practices which therefore warrants the use of interventions to improve their practices.^18^-^19^

Our results, that health and sanitary workers had significantly improved levels of knowledge, attitude and practices after the training, are similar to existent literature which shows that such an intervention is useful. A study from Sudan measured similar outcomes and showed the persistence of the effects of training of healthcare workers on HCWM guidelines after three months.^20^

Research in the past have consistently indicated the importance of trainings and knowledge improvement of health as well as sanitary workers as a pivot to improve HCWM practices.^21^ Others have identified training as part of intervention package to improve the HCWM related practices in these countries.^22^ Our results show a persistence of significant difference in the knowledge, attitude and practices among the group which received training as compared to control. The intervention group had better knowledge, positive attitudes and good practices. The sustainability of the high scores in the knowledge, attitude and practice composite scores in the intervention group after 18 months suggests the intervention was highly effective.

We also found that there was no statistically significant change in scores for both health and sanitary workers from first follow up to final follow up with respect to Knowledge, attitude or practices. This could be explained by the fact that these workers has already achieved very high level of scores in individual items of HCWM related guidelines that they did not improve significantly from the previous measurement; though as discussed they increased adequately after the training. This also reflects upon the importance of the regular refresher trainings for these workers in order to further improve their practices.

## CONCLUSION

We conclude that effects of trainings of health and sanitary workers on HCWM guidelines persist and are sustainable among the intervention group even after the passage of about one and a half year which is sufficient to measure the impact of such an intervention. We recommend that the training as an intervention be included in the overall health care related policies especially which regulate the public and private sector hospitals. Implementation of trainings for the health and sanitary workers in hospitals may require uniform guidelines tailored to local setting with regular follow up for improving HCWM and therefore the quality of health services. Our results may be generalized to other similar settings in the region and elsewhere. We also recommend for further research with more robust designs like; Randomized control trials to generate more reliable evidence.

### Limitations of the study

Hospital based study has some issue of generalizability. Hence, the impact of interventions may be applied at every level of healthcare facility in the country.
